# Exploiting the Property of a New Series of 1,2,3-Triazole Compounds as Inhibitors of In Vivo and In Vitro Toxic Effects Caused by *Bothrops jararacussu* Snake Venom

**DOI:** 10.3390/tropicalmed11080224

**Published:** 2026-08-11

**Authors:** Aldo Rodrigues da Silva, Ana Cláudia Rodrigues da Silva, Eladio Flores Sanchez, Gabriel Alves Souto de Aquino, Vitor Francisco Ferreira, Sabrina Baptista Ferreira, André Lopes Fuly

**Affiliations:** 1 Instituto de Biologia, Universidade Federal Fluminense, Niterói 24020–141, RJ, Brazil; biof.aldo@yahoo.com.br (A.R.d.S.); anacrs1@yahoo.com.br (A.C.R.d.S.); 2 Fundação Ezequiel Dias, Belo Horizonte 30510–010, MG, Brazil; eladio.flores@funed.mg.gov.br; 3 Departamento de Química Orgânica, Instituto de Química, Universidade Federal do Rio de Janeiro, Rio de Janeiro 21949-900, RJ, Brazil; gabrielalves.aq@gmail.com (G.A.S.d.A.); sabrinab@iq.ufrj.br (S.B.F.); 4 Faculdade de Farmácia, Laboratório Inovação em Química e Tecnologia Farmacêutica, Universidade Federal Fluminense, Niterói 24220-900, RJ, Brazil; vitorferreira@id.uff.br

**Keywords:** triazole scaffold, antivenom, *B. jararacussu* snake, treatment, neglected disease

## Abstract

(1) Background: Snake bite envenomation is a neglected disease that affects impoverished and rural areas, causing deaths and physical sequelae. (2) Objective: A novel series of eight 1,2,3-triazole compounds, AM50, AM51, AM52, AM53, AM54, AM55, AM56, and AM57, were synthesized and assessed as inhibitors of toxic activities of *B. jararacussu* venom. Methods: *B. jararacussu* venom was pre-incubated with each of the compounds and after the coagulant, proteolytic, hemorrhagic, edematogenic, and lethal activities were assessed. The structure of compounds was analyzed by NMR and FT-IR spectroscopy techniques, and toxicity was predicted through OSIRIS and SwissADME. (3) Results: AM56 and AM57 inhibited the plasma coagulant and prevented hemorrhagic activity of the venom. Proteolysis and hemorrhage were inhibited by AM52, AM54, AM55, and AM56 (20–30%), as well as AM50, AM51, and AM57 (30–60%). AM57 fully protected the mice from death caused by venom, and AM50, AM53–AM57 inhibited edema of venom by 10–20%; AM51 and AM52 did not inhibit edema. In silico analysis of compounds revealed satisfactory parameters for drug discovery. (4) Conclusions: 1,2,3–triazole compounds inhibited the major toxic activities of *B. jararacussu* venom and should be considered as a lead for further investigation as adjunct of antivenom therapeutics.

## 1. Introduction

Snakebite envenomation (SBE) is a neglected public health problem of low and middle-income tropical and subtropical developing and impoverished regions [1,2]. According to the World Health Organization (WHO), approximately 1.8–2.7 million incidents, 130,000 deaths, and 400,000 physical sequelae, including amputations and disabilities, occur annually worldwide, with Asia, Africa, and Latin America showing the highest epidemiological rates. However, it is well established that these data are underreported, and it is expected that the numbers are three times higher [2,3]. Besides the high morbidity and physical disabilities rates of SBE, economic, social, and psychiatric (including mental and post-traumatic stress disorders) consequences should also be considered [4,5,6].

Brazil has a rich biodiversity containing approximately 400 species of snakes, of which roughly 70 are venomous, distributed in two families: Elapidae and Viperidae [7]. The former comprises the genera *Micrurus* and *Leptomicrurus*, popularly known as “corais–verdadeiras”, which are widely distributed in Brazil, with approximately 40 species, reaching 20 to 100 cm in length. They cause less than 1% of SBE with predominantly neurotoxic symptoms [1]. Elapidae is also endemic to Asia, Australia, Africa, and Americas, and some marine species may occur in the Pacific and Indian Oceans [8]. Viperidae, known as vipers, are found in most parts of the world, except for Antarctica and other isolated islands [3]. In Brazil, vipers are considered the largest family, with approximately 40 species that are responsible for 98% of the registered SBE [9], and are thus considered the most venomous snakes. They have the most developed and specialized venom-injecting apparatus, as well as an intriguing and complex venom composition. In Brazil, vipers have three major genera, including *Crotalus* (“cascavéis” or rattlesnakes), *Lachesis* (“surucucu” or bushmasters), and *Bothrops* (jararacas) [9], of which the latter genus has the highest rate of incidents, ca. 88%, in Brazil [9]. Among *Bothrops*, species *B. jararaca*, *B. moojeni*, and *B. jararacussu*, *B. atrox* may be considered the major clinical species in Brazil, due to epidemiological and medical issues, as well as in other countries of South America, including Paraguay, Argentina, and Bolivia [3,10]. In Brazil, from 2011 to 2020, of 1.9 million incidents with snakes, at least 1.7 million were caused by unidentified snakes and 235,872 by venomous snakes, with *Bothrops* spp. as the major prominent group [11].

*B. jararacussu* demonstrates very aggressive behavior and is a large and robust venomous species, growing up to 2 m in length, with large fangs of 2.5 cm that allow injections of huge amounts of venom [3]. In Brazil, *B. jararacussu* is found in the coastal areas of Bahia to Santa Catarina, as well as in the Central region of Brazil, including Mato Grosso do Sul and Goiás [3,9,12]. In general, envenomation caused by *Bothrops* has similar clinical and symptomatic consequences, including systemic and local hemorrhage, intense muscle damage, hemolysis, kidney, respiratory, and cardiac failure, disturbances in blood coagulation, edema, and eventually death. Snake venoms contain active compounds, including snake venom metalloproteinases (SVMPs), snake venom serine proteinases (SVSPs), phospholipases A_2_ (PLA_2_s), L-aminoacid oxidases (LAAOs), and hyaluronidases [11,13,14]. In fact, snake venoms are a cocktail of enzymes (approximately 95–99%) that are responsible for immobilization and digestion of prey, as well as for the toxic effects listed above. In addition, lower quantities of inorganic compounds (such as Na^+^, Zn^+2^, Ca^+2^, and Mg^+2^) are also present in the venoms and may act as cofactors to the active components [13,15].

Snake antivenom immunoglobins, also called antivenom, antivenins, or antisnake venoms, are the primary available therapeutics, recognized by the World Health Organization (WHO) and worldwide Ministries of Health for the treatment of SBE [16,17]. In 2008, the WHO approved the first edition of the guidelines for the production, control, and regulation of antivenoms [18], and in 2017, the WHO designated SBE as a Category A neglected tropical disease (NTD), thereby giving it global health priority [19]. Antivenoms are administered intravenously to victims in hospitals and effectively prevent death, but they are ineffective against the local toxic effects, including muscle damage and edema, that contribute to physical sequelae of the affected limb [20]. Moreover, the efficacy of neutralizing antivenoms depends on the early administration, dosage, venom variability and diversity, as well as other ecological variations [2,5,21]. Undoubtedly, it has been a challenge in manufacturing antivenoms, and few companies, governments, and public institutions have capacity to produce antivenoms of high or adequate quality to make immunogens affordable for people. Nevertheless, defining new therapeutics or compounds as potential adjunctive candidates is a challenge, and they are of great interest to society for overcoming the disadvantages of antivenoms [22].

Triazoles are organic heterocyclic compounds, with a five-membered ring structure containing two carbon atoms and three nitrogen atoms, and have been widely used in drug design and novel drug discovery [23,24]. There are two types of triazoles, 1,2,3-triazole and 1,2,4-triazole, with the former being widely used in drug development. Many triazole-based compounds have been synthesized [23,25] and widely explored due to their wide range of pharmacological effects, including antifungal [26], anticoagulant and antiplatelet aggregation [27], anticancer [28], antimalarial [29], and antivenom [30,31,32]. It is worth noting that the triazole scaffold with different substituents appended to the triazole core influences critical pharmacological parameters [25,33,34]. Some commercial drugs contain the triazole moiety, including Voriconazole and Fluconazole (antifungal) and Ribavirin (antiviral) [24]. Therefore, triazoles have favorable features in drug research, including low toxicity and good pharmacokinetics, are the most stable among azoles, and have contributed to treatment of human diseases over recent decades [24,34].

Therefore, in this work, to develop the use of triazole scaffolds as antivenoms, a novel series of eight 1,2,3-triazole compounds, AM50, AM51, AM52, AM53, AM54, AM55, AM56, and AM57, was synthesized and assessed as inhibitors of the selected toxic activities of *B. jararacussu* venom, including plasma coagulant, proteolytic, hemorrhagic, edematogenic, and lethal.

## 2. Materials and Methods

### 2.1. Snake Venom, Animals, and Chemical Supplies

Lyophilized *B. jararacussu* venom was supplied by the Ezequiel Dias Foundation, Belo Horizonte, MG, Brazil, and stored at −20 °C prior to use. The venom was dissolved in physiological saline to a concentration of 1 mg/mL. The collection of venom and maintenance of snakes in captivity complied with the current legislation, under the authorization of the Brazilian System for Management Genetic of Heritage and Associated Traditional Knowledge (SISGEN), with protocols A39CD4E and A24A925. Swiss mice (18–20 g) were obtained from the Laboratory Animal Care of Federal Fluminense University (UFF) and housed under constant light (12 h light/dark cycle) and temperature (24 ± 1 °C) with water *ad libitum*. In vivo experiments were conducted according to the guidelines of the Brazilian College for Animal Experimentation (COBEA) and were approved by the Ethics Committee on the Use of Animals of UFF with the identification protocol number 297, approved December 2012. Solvents were of the best grade available.

### 2.2. Chemistry of the 1,2,3-Triazole Compounds

**Scheme 1:**
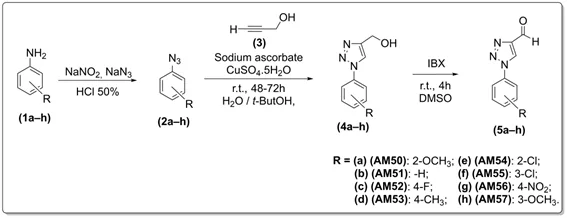
Synthetic route for the preparation of 1,2,3-triazole compounds AM50, AM51, AM52, AM53, AM54, AM55, AM56, and AM57.

The synthesis of the compounds followed the established methodology described previously [35], which we have used before [36,37]. The methodology for preparing compounds (5a–h) began with the synthesis of aromatic azides from commercial anilines via diazotization with sodium nitrite, followed by addition of sodium azide, yielding the organic azides (2a–h) as brown oils of 60–100%. The azides were used directly in the next step without purification to prevent degradation. Then, the synthesis of compounds (4a–h) was performed through a dipolar 1,3–cycloaddition reaction using sodium ascorbate and copper (II) sulfate, with reaction times varying between 48 h and 72 h. The reaction was carried out in the dark to avoid degradation of the azides and oxidation of sodium ascorbate, using a mixture of equal volumes of t-BuOH and water as a solvent (Scheme 1). The compounds were obtained as white or yellow solids with yields ranging from 30% to 82%, and their structures were confirmed by Fourier transform infrared (FTIR) and ^1^H and ^13^C nuclear magnetic resonance (NMR) spectroscopy techniques. The final products (5a–h) were obtained by oxidizing the primary alcohol to an aldehyde using 2-iodoxibenzoic acid (IBX) and dimethyl sulfoxide (DMSO) as solvents (Scheme 1). The aldehydes were obtained as white or light-yellow solids in yields ranging from 55% to 100%, and their structures are consistent with those described in the literature.

### 2.3. General Procedure for Preparation of Compounds (2a–h)

Azide compounds were obtained according to a previously reported method in [35]. In an Erlenmeyer flask containing 1 mmol of aromatic amine (1a–h), 1 mL of 6 M hydrochloric acid solution (50%) was added in an ice bath (maintaining temperature between 0 and 5 °C). Then, an aqueous solution of 1.5 mmol of sodium nitrite (NaNO_2_) in 2.5 mL of water was slowly added under vigorous stirring. Thereafter, stirring continued at low temperature for 30 min. Subsequently, a solution of 4 mmol of sodium azide (NaN_3_) in 5 mL of water was slowly added while maintaining the temperature between 0 and 5 °C. Lastly, the reaction was kept at room temperature for the necessary time until it was completed. Then, the mixture was extracted with ethyl acetate, and the organic phase was washed with saturated sodium bicarbonate solution and water and dried over anhydrous sodium sulfate. The solvent was evaporated under reduced pressure to obtain the aromatic azides (2a–h). The residual crude product was used directly without purification.

### 2.4. General Procedure for Preparation of (4a–h)

To a solution containing 1 mmol of propargyl alcohol (3) in t-BuOH (1 mL) and water (1 mL), 1.5 mmol of the appropriate aromatic azide (2a–h), 0.1 mmol of CuSO_4_·5H_2_O, and 0.2 mmol of sodium ascorbate were added. The resulting suspension was kept at room temperature for 48–72 h. After this time, the mixture was diluted with 5 mL each of dichloromethane and water. The organic phases were separated, dried with anhydrous sodium sulfate, and concentrated at reduced pressure. The crude material was purified via silica gel column chromatography using a gradient of hexane–ethyl acetate to furnish the 1,2,3–triazole compounds (4a–h).

1-(2-Methoxyphenyl)-1H-1,2,3–triazole-4-yl-methanol (4a)

White solid, mp = 116–117 °C. IR νmax (cm^−1^): 3276, 3123, 3078, 2954, 1603, 1509, 1472, 1286, 1250, 1180, 1122, 1016, 861, 761, 715, 682. ^1^H NMR (DMSO-d_6_, 300 MHz): δ 3.58 (3H, s, CH_3_), 4.95 (2H, d, J = 5.4, CH_2_OH), 2.82 (1H, t, J = 5.4, OH), 7.40–7.49 (4H, m, H-3′, H-4′, H-5′, and H-6′), 8.15 (1H, s, H-5). ^13^C NMR (DMSO-d_6_, 75 MHz): δ 59.8 (C- 2′), 59.9 (OCH_3_), 126.6 (C-5), 127.2 (C-3′), 128.3 (C-4′), 131.0 (C-5′), 131.9 (C-6′), 133.8 (C-6), 137.6 (C-1′), 150.2 (C-4).

1-(Phenyl)-1H-1,2,3–triazole-4-yl-methanol (4b)

White solid, mp = 109–110 °C. IR νmax (cm^−1^): 3379, 3138, 1594, 1501, 1465, 1349, 1238, 1176, 1012, 819, 758, 684. ^1^H NMR (DMSO-d_6_, 300 MHz): δ 4.73 (1H, d, J = 5.6, OH), 5.46 (2H, td, J = 5.6 and 1.7, CH_2_OH), 7.56–7.62 (1H, m, H-4′), 7.68–7.73 (2H, m, H-3′ and H-5′), 8.00–8.03 (2H, m, H-2′ and H-6′), 8.77 (1H, s, H-5). ^13^C NMR (DMSO-d_6_, 75 MHz): δ 55.7 (CH_2_OH), 120.6 (C-4′), 121.6 (C-5), 129.1 (C-3′ and C-5′), 130.5 (C-2′ and C-6′), 137.4 (C-1′), 149.8 (C-4).

1-(4-Fluorophenyl)-1H-1,2,3–triazole-4-yl-methanol (4c)

White solid, mp = 133–134 °C. IR νmax (cm^−1^): 3397, 3230, 3066, 1575, 1494, 1290, 1250, 1060, 824, 685. ^1^H NMR (DMSO-d_6_, 300 MHz): δ 4.90 (2H, d, J = 5.8, CH_2_OH), 5.50 (1H, t, J = 5.8, OH), 7.72 (2H, d, J = 9.3, H-3′ and H-5′), 8.03 (2H, d, J = 9.3, H-2′ and H-6′), 8.80 (1H, s, H-5). ^13^C NMR (DMSO-d_6_, 75 MHz): δ 56.0 (CH_2_OH), 121.3 (C-5), 121.8 (C-2′ and C-6′), 130.2 (C-2′ and C-6′), 141.6 (C-1′), 142.4 (C-4), 160.8 (C-4′).

1-(4-Methylphenyl)-1H-1,2,3–triazole-4-yl-methanol (4d)

White solid, mp = 124–125 °C. IR νmax (cm^−1^): 3426, 3221, 3117, 1517, 1239, 1187, 1042, 1013, 824, 772, 714, 686. ^1^H NMR (DMSO-d_6_, 300 MHz): δ 2.28 (3H, s, CH_3_), 4.66 (2H, d, J = 5.2, CH_2_OH), 5.48 (1H, s, OH), 7.65 (2H, d, J = 9.5, H-3′ and H-5′), 7.99 (2H, d, J = 9.5, H-2′ and H-6′), 8.89 (1H, s, H-5). ^13^C NMR (DMSO-d_6_, 75 MHz): δ 21.1 (CH_3_), 59.0 (CH_2_OH), 120.0 (C-5), 121.6 (C-2′ and C-6′), 130.9 (C-2′ and C-6′), 132.7 (C-4′), 135.3 (C-1′), 150.4 (C-4).

1-(2-Chlorophenyl)-1H-1,2,3–triazole-4-yl-methanol (4e)

White solid, mp = 86–87 °C. IR νmax (cm^−1^): 3266, 3112, 3072, 2988, 2935, 2868, 1498, 1467, 1420, 1380, 1341, 1227, 1181, 1122, 1041, 1012, 947, 882, 765, 675. ^1^H NMR (DMSO-d_6_, 300 MHz): δ 4.62 (2H, d, J = 5.6, CH_2_OH), 5.32 (1H, t, J = 5.6, OH), 7.55–7.67 (3H, m, H-3′, H-4′ and H-5′), 7.76 (1H, dd, J = 1.95 and 1.22), 8.40 (1H, s, H-5). ^13^C NMR (DMSO-d_6_, 75 MHz): δ 128.4 (C-2′), 124.8 (C-5), 131.4 (C-3′), 130.5 (C-4′), 128.3 (C-5′), 128.2 (C-6′), 54.8 (C-6), 135.0 (C-1′), 148.0 (C-4).

1-(3-Chlorophenyl)-1H-1,2,3–triazole-4-yl-methanol (4f)

White solid, mp = 95–97 °C. IR νmax (cm^−1^): 3263, 3090, 1593, 1487, 1464, 1236, 1043, 871, 784, 671. ^1^H NMR (DMSO-d_6_, 300 MHz): δ 4.61 (2H, d, J = 4.7, CH_2_OH), 5.36 (2H, t, J = 5.5, OH), 7.54–7.56 (1H, m, H-4′), 7.62 (1H, t, J = 8 Hz, H-5′), 7.91–7.94 (1H, m, H-6′), 8.04–8.06 (1H, m, H-2′), 8.77 (1H, s, H-5). ^13^C NMR (DMSO-d_6_, 75 MHz): δ 54.9 (CH_2_OH), 118.4 (C-5′), 119.6 (C-4′), 121.1 (C-5), 128.2 (C-2′), 131.6 (C-6′), 134.1 (C-3′), 137.7 (C-1′), 149.3 (C-4).

1-(4-Nitrophenyl)-1H-1,2,3–triazole-4-yl-methanol (4g)

White solid, mp = 201–202 °C. IR νmax (cm^−1^): 3283, 3138, 3080, 2934, 1583, 1484, 1452, 1369, 1237, 1094, 1054, 1022, 844, 808. ^1^H NMR (DMSO-d_6_, 300 MHz): δ 4.63 (2H, s, CH_2_OH), 5.39 (1H, s, OH), 8.23 (1H, d, J = 9.1, H-2′), 8.44 (1H, d, J = 9.1, H-3′), 8.23 (1H, d, J = 9.1, H-5′), 8.44 (1H, d, J = 9.1, H-6′). ^13^C NMR (DMSO-d_6_, 75 MHz): δ 54.2 (CH_2_OH), 120.7 (C-5), 119.7 (C-2′), 119.7 (C-6′), 140.3 (C-4′), 124.9 (C-3′), 124.9 (C-5′), 145.9 (C-1′), 149.2 (C-4).

1-(3-Methoxyphenyl)-1H-1,2,3–triazole-4-yl-methanol (4h)

White solid, mp = 100–102 °C. IR νmax (cm^−1^): 3256, 3110, 3070, 2987, 2930, 2868, 1488, 1447, 1419, 1380, 1351, 1227, 1171, 1112, 1041, 1012, 947, 892, 766, 709, 665, 643. ^1^H NMR (DMSO-d_6_, 300 MHz): δ 3.87 (3H, s, CH^3^), 4.88 (2H, d, J = 5.7, CH_2_OH), 2.9 (1H, t, J = 5.7, OH), 7.32 (1H, dd, J = 2.3 H-2′), 6.97 (1H, ddd, J = 8.1, J = 2.3, J = 0.9, H-4′), 7.40 (1H, dd, J = 8.1, H-5′), 7.23 (1H, ddd, J = 8.1, J = 2.3, J = 0.9, H-6′), 7.97 (1H, s, H-5). ^13^C NMR (DMSO-d6, 75 MHz): δ 55.5 (OCH3), 56.1 (CH2), 112.3 (C-4′), 114.6 (C-6′), 120.2 (C-5), 121.9 (C-2′), 130.4 (C-5′), 137.9 (C-1′), 148.4 (C-4), 160.5 (C-3′).

### 2.5. General Procedure for Preparation of Compounds 5a–h (AM50–AM57)

In a round-bottom flask containing 1 mmol of (3a–h), 2.75 mL of DMSO was added, followed by the addition of 1.1 mmol of IBX, and stirred at room temperature for 4 h. Then, H_2_O (20 mL) was added to precipitate IBX crystals, and these crystals were decanted. The mother liquor was extracted with ethyl acetate, washed with NaHCO_3_ solution, and dried over MgSO_4_ to obtain pure aldehydes.

1-(2-Methoxyphenyl)-1H-1,2,3–triazole-4-carbaldehyde (5a–AM50)

White solid, mp = 101–105 °C. IR νmax (cm^−1^): 3420, 3100, 3040, 2997, 1693, 1602, 1501, 1260, 1171, 1040, 840, 773. 1H NMR (DMSO-d_6_, 300 MHz): δ 3.91 (3H, s, OCH_3_), 7.90–7.95 (4H, m, H-2′, H-4′, H-5′ and H-6′), 9.65 (1H, s, H-5), 10.00 (1H, s, H-6). ^13^C NMR (DMSO-d6, 75 MHz):δ57.8 (CH_3_), 121.5 (C-2′), 123.3 (C-6′), 125.1 (C-5), 116.8 (C-5′), 148.2 (C-4′), 115.8 (C-3′), 131.2 (C-1′), 160.6 (C-4), 190.8 (C-6).

1-Phenyl-1H-1,2,3–triazole-4-carbaldehyde (5b–AM51)

White solid, mp = 95–96 °C. IR νmax (cm^−1^): 3431, 3131, 1691, 1529, 1209, 1168, 990, 853, 782, 761, 683. ^1^H NMR (DMSO-d_6_, 300 MHz): δ 7.50–7.79 (4H, m, H-2′, H-3′, H-5′ and H-6′), 8.54 (1H, s, H-5), 10.23 (1H, s, H-6). ^13^C NMR (DMSO-d_6_, 75 MHz): δ 120.8 (C-2′ and C-6′), 123.1 (C-5), 129.7 (C-4′), 130.0 (C-3′ and C-5′), 136.1 (C-1′), 148.0 (C-4), 185.0 (C-6), MS (ESI) m/z 173 (M)+.

1-(4-Fluorophenyl)-1H-1,2,3–triazole-4-carbaldehyde (5c–AM52)

Yellow solid, mp = 127–128 °C. IR νmax (cm^−1^): 3382, 3116, 3046, 1702, 1513, 1231, 1214, 1009, 837, 780, 613. ^1^H NMR (DMSO-d_6_, 300MHz): δ 7.80 (2H, d, J = 9.5, H-3′ and H-5′), 8.10 (2H, d, J = 9.5, H-2′ and H-6′), 8.85 (1H, s, H-5), 10,20 (1H, s, H-6). ^13^C NMR (DMSO-d_6_, 75 MHz): δ 119.8 (C-2′ and C-6′), 121.1 (C-5), 129.7 (C-4′), 131.0 (C-3′ and C-5′), 140.1 (C-1′), 150.0 (C-4), 183.0 (C-6). MS (ESI) m/z 191 (M)+.

1-(4-Methyl)-1H-1,2,3–triazole-4-carbaldehyde (5d–AM53)

White solid, mp = 105–107 °C. IR νmax (cm^−1^): 3120, 3090, 2924, 2874, 1693, 1534, 1490, 1406, 1265, 858, 775. ^1^H NMR (DMSO-d_6_, 300 MHz): 7,48 (1H, d, J = 9.4, H-5′ e H-3′); 7.55 (1H, d, J = 9.4, H-2′ e H-6′); 9.78 (1H, s, H-5); 10.10 (1H, s, CHO). ^13^C NMR (DMSO-d_6_, 75 MHz): 40.00 (CH3); 122.1 (C-2′); 122.6 (C-6′); 127.4 (C-5); 126.1 (C-5′); 147.8 (C-4′); 125.4 (C-3′); 140.9 (C-1′); 148.4 (C-4); 183.5 (C-6).

1-(2-Chlorophenyl)-1H-1,2,3–triazole-4-carbaldehyde (5e–AM54)

Yellow solid, mp = 76–77 °C. IR νmax (cm^−1^): 3138, 3092, 2860, 1704, 1573, 1529, 1487, 1402, 1371, 1261, 1199, 1170, 1142, 1100, 1075, 1042, 982, 857, 816, 698, 649. ^1^H NMR (DMSO-d_6_, 300 MHz): δ 7.52–7.82 (4H, m, H-3′,H-4′,H-5′ and H-6′), 9.34 (1H, s, H-5), 10.13 (1H, s, CHO). ^13^C NMR (DMSO-d_6_, 75 MHz): δ 131.8 (C-2′), 126.6 (C-6′), 128.6 (C-5), 126.7 (C-5′), 128.7 (C-4′), 130.4 (C-3′), 126.8 (C-1′), 144.9 (C-4), 182.9 (C-6).

1-(3-Chlorophenyl)-1H-1,2,3–triazole-4-carbaldehyde (5f–AM55)

Yellowwish solid, mp = 89–92 °C. IR νmax (cm^−1^): 3140, 3091, 2865, 1703, 1490, 1410, 1172, 1150, 1100, 1085, 1042, 850, 820, 680. ^1^H NMR (DMSO-d6, 300 MHz): δ 7.50–7.62 (4H, m, H-3′,H-4′,H-5′ and H-6′), 9.30 (1H, s, H-5), 10.23 (1H, s, CHO). ^13^C NMR (DMSO-d_6_, 75 MHz): δ 132.8 (C-2′), 130.6 (C-6′), 129.4 (C-5), 126.7 (C-5′), 127.7 (C-4′), 131.3 (C-3′), 127.8 (C-1′), 145.9 (C-4), 180.0 (C-6).

1-(4-Nitro)-1H-1,2,3–triazole-4-carbaldehyde (5g–AM56)

Yellow solid, mp = 110–113 °C. IR νmax (cm^−1^): 3127, 3088, 2924, 2873, 1693, 1531, 1487, 1437, 1404, 1261, 857, 822, 771. ^1^H NMR (DMSO-d_6_, 300 MHz): 8.3 (1H, d, J = 9.4, H-6′); 8.48 (1H, d, J = 9.4, H-5′ e H-3′); 8.3 (1H, d, J = 9.4, H-2′ e H-6′); 9.75 (1H, s, H-5); 10.14 (1H, s, CHO). ^13^C NMR (DMSO-d_6_, 75 MHz): 121.1 (C-2′); 121.1 (C-6′); 126.4 (C-5); 125.1 (C-5′); 146.9 (C-4′); 125.1 (C-3′); 139.9 (C-1′): 147.4 (C-4); 184.5 (C-6).

1-(3-Methoxyphenyl)-1H-1,2,3–triazole-4-carbaldehyde (5h–AM57)

White solid, mp = 106–107 °C. IR νmax (cm^−1^): 3427, 3106, 3038, 2998, 1690, 1607, 1503, 1259, 1169, 1042, 845, 773, 677. ^1^H NMR (DMSO-d_6_, 300 MHz): δ 3.90 (3H, s, OCH3), 7.88–7.95 (4H, m, H-2′, H-4′, H-5′ and H-6′), 9.65 (1H, s, H-5), 10.30 (1H, s, H-6). ^13^C NMR (DMSO-d6, 75 MHz):δ56.8 (CH3), 121.3 (C-2′), 122.3 (C-6′), 125.0 (C-5), 115.8 (C-5′), 148.0 (C-4′), 114.8 (C-3′), 130.2 (C-1′), 160.8 (C-4), 185.8 (C-6).

After synthesis, compounds AM50–AM57 were dissolved in DMSO, supplied from Sigma Chemical Co., (St. Louis, MS, USA), to a concentration of 10 mg/mL and stored at −20 °C, prior to determining their effects on in vitro and in vivo toxic activities.

### 2.6. Effect of 1,2,3-Triazoles on the Plasma Coagulation of B. Jararacussu Venom

The coagulant activity of *B. jararacussu* venom was assessed using a pool of human plasma kindly supplied from the blood bank of the Antônio Pedro Hospital of Federal Fluminense University (HUAP–UFF), under the authorization of the Committee for Ethical in Experimentation (CEP) of UFF. An amount of 200 μL of plasma (previously diluted 1:1, vol/vol in physiological saline) was kept for 1 min at 37 °C, followed by the addition of different concentrations of *B. jararacussu* venom (5–50 μg/mL), yielding a final volume of 250 μL. Then, the plasma coagulation was monitored in seconds via a digital coagulometer (model Amelung KC4A, Labcom, Germany). The amount of venom (μg/mL) able to clot plasma at approximately 60 s was defined as the minimum coagulation concentration (MCC) [38]. One arbitrary unit MCC (62 μg/mL) was incubated for 30 min at 25 °C with physiological saline, DMSO (0.9% *v*/*v*, final concentration), as well as compounds AM50–AM57 (at the ratio of 1:5 venom:compound (wt/wt)). After incubation, 50 μL of each mixture was added to plasma and coagulation was monitored, as described above. Negative controls were contained in the medium-reaction-only compounds, physiological saline, or DMSO in the absence of venom. Each experimental group was performed with four replicates of two individual experiments, yielding *n* = 8.

### 2.7. Effect of 1,2,3-Triazoles on the Proteolytic Activity of B. Jararacussu Venom

The proteolytic activity of *B. jararacussu* venom was determined using azocasein (from Sigma Chemical Co., (St. Louis, MS, USA) as the proteinaceous substrate [39]. Different concentrations of *B. jararacussu* venom (2–20 μg/mL) were incubated for 90 min at 37 °C with 0.2% azocasein (*w*/*v*) dissolved in 20 mM Tris-HCl, 8 mM CaCl_2_, pH 8.8. Enzymatic activity was stopped by adding 10% trichloroacetic acid (TCA), followed by centrifugation of the tubes for 3 min at 16,740 relative centrifugal force (RFC). Then, 1 mL of the supernatant was transferred to tubes containing 2 M NaOH, and the samples were read using a spectrophotometer at the absorbance of 420 nm. The concentration of *B. jararacussu* venom (μg/mL) able to produce absorbance values of ~0.2, which corresponds to the maximal velocity of reaction, was defined as the arbitrary unit enzymatic unit (EU). One EU of *B. jararacussu* venom (15 μg/mL) was incubated with compounds AM50–AM57 at the ratio of 1:5 (venom:compound, wt/wt), with physiological saline and DMSO for 30 min at 25 °C. Then, an aliquot (50 μL) was added to the medium reaction, and the proteolytic activity was performed, as described above. Negative controls were contained in the medium-reaction-only compounds, physiological saline, or DMSO in the absence of venom. Each experimental group was performed with four replicates of two individual experiments, yielding *n* = 8.

### 2.8. Effect of 1,2,3-Triazoles on the Hemorrhagic Activity of B. Jararacussu Venom

The hemorrhagic activity of *B. jararacussu* venom was assessed using the procedure of [40] with modifications. *B. jararacussu* venom was injected intradermally (i.d.) into the abdominal skin of the mice, and 2 h later, animals were euthanized and the abdominal skin was removed, stretched, and inspected for visual changes in the internal aspects to localize hemorrhagic spots. The arbitrary unit, minimum hemorrhagic dose (MHD), was defined as the dose of *B. jararacussu* venom (μg/mouse) able to produce a hemorrhagic halo of approximately 10 mm (mm). Antihemorrhagic effect of compounds AM50–AM57 on *B. jararacussu* venom-induced hemorrhage was assessed by incubating 2 MHD of venom (64 μg/mouse) with compounds (at the ratio of 1:5 venom:compound, wt/wt) and solvents (physiological saline and DMSO) for 30 min at 25 °C. Then, a 100 μL aliquot of each mixture was injected i.d. into the mice, and the hemorrhagic activity was assessed. In parallel, compounds, saline, or DMSO were injected i.d. in the absence of venom. The total volume injected into the mice was 100 μL. Each experimental group was performed with three replicates of two individual experiments, yielding *n* = 6.

### 2.9. Effect of 1,2,3-Triazoles on the Edematogenic Activity of B. Jararacussu Venom

The edematogenic activity of *B. jararacussu* venom was determined according to the method of [41], with modifications. Mice received a single subcutaneous (s.c.) injection of *B. jararacussu* venom mixed with physiological saline into the right paw (positive controls), while the left received an injection of physiological saline or DMSO (negative controls) in the absence of venom. After 1 h of injection, the mice were euthanized, their paws were cut at the ankle joint and weighed, and edema was expressed as the percentage of increase in the weight of the right paw relative to the left. *B. jararacussu* venom (16 μg/mouse) was mixed with compounds AM50–AM57, at the 1:5 ratio of venom:compound (wt/wt) and solvents (physiological saline and DMSO) for 30 min at 25 °C; then, each mixture was injected s.c. into animals and edema was evaluated, as described. Control mice received injections of compounds or solvents in the absence of venom. The total volume injected into the mice was 50 μL. Each experimental group was performed with two replicates of three individual experiments, yielding *n* = 6.

### 2.10. Effect of 1,2,3-Triazoles on the Lethal Activity of B. Jararacussu Venom

Intraperitoneal (i.p.) injection of *B. jararacussu* venom (130 μg/mouse) mixed with solvents physiological saline or DMSO killed mice approximately at 60 min. This dose of *B. jararacussu* venom was incubated with compounds AM50–AM57, at the ratio of 1:5 venom:compound and with solvents for 30 min at 25 °C, followed by i.p. injection of samples into the mice. Then, the deaths of the mice were recorded. As negative controls, the mice received injections of compounds and solvents (physiological saline and DMSO) in the absence of *B. jararacussu* venom. The total volume injected into the mice was 100 μL, and the maximal survival time was 360 min. After this period of time, the surviving animals were euthanized by overdose of isoflurane via gauze. Each experimental group was performed with two replicates of three individual experiments, yielding *n* = 6.

### 2.11. Prediction of Pharmacological and Toxicological Properties by Admetsar

The theoretical study of the toxicity of compounds AM50–AM57 was predicted using the open-access program OSIRIS^®^ Property Explorer (http://www.organic-chemistry.org/prog/peo/, accessed on 20 October 2021) which analyzed, based on the chemical structure of each compound, four toxic parameters, including mutagenicity, tumorigenicity, skin irritability, and negative effects on reproduction. Moreover, via the online ADMETsar tool (http://pubs.acs.org/doi/abs/10.1021/ci100104j, accessed on 15 March 2022), the parameters of absorption, distribution, metabolism, excretion, and toxicity (ADMET), solubility, drug score, druglikeness, clog P, LogS, molecular weight (MW), number of hydrogen bond acceptor (nHBA), and donors (nHBD) were evaluated.

### 2.12. Statistical Analysis

The results are expressed as the means ± standard deviation (SD) and analyzed via ANOVA, followed by Dunnet’s post hoc test, and *p* values < 0.05 were considered significant. The graphs were generated using Graph Prism^®^ program version 9.0 (Software Inc., San Diego, CA, USA).

## 3. Results

### 3.1. Effect of 1,2,3-Triazole Compound on In Vitro Activities of B. Jararacussu Venom

**Figure 1:**
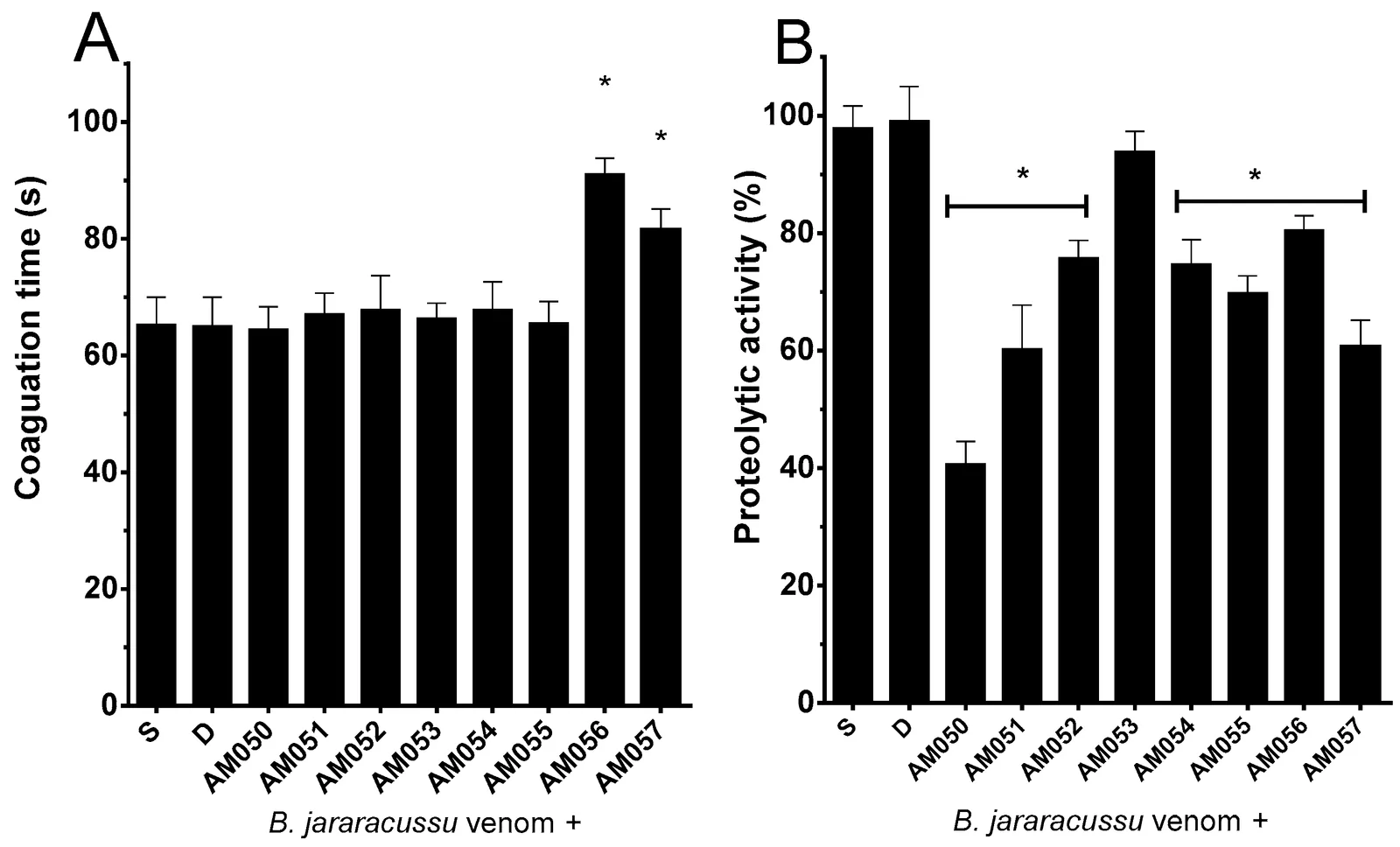
*B. jararacussu* venom was incubated with saline solution (S), DMSO (D), and compounds AM50, AM51, AM52, AM53, AM54, AM55, AM56, and AM57 for 30 min at 25 °C; then, the plasma coagulation time (**A**) and the proteolytic activity (**B**) were determined, as described in the Methods section. The results are expressed as the means ± SD (*n* = 8). * *p* < 0.05 compared with *B. jararacussu* venom + S and *B. jararacussu* venom + D.

The positive control group consisted of one MCC of *B. jararacussu* venom (62 μg/mL) mixed with physiological saline solution, which clotted the plasma at approximately 65 s (Figure 1A); DMSO did not interfere with the coagulant of *B. jararacussu* venom (Figure 1A). Then, one MCC of *B. jararacussu* venom (62 μg/mL) was incubated with each of the compounds, AM50–AM57 (310 μg/mL), and these were added to the plasma. As seen in Figure 1A, compounds AM56 and AM57 delayed the plasma coagulation times of venom to approximately 91 s and 81 s, respectively, thus inhibiting such activity of *B. jararacussu* venom. Despite the moderate light inhibitory effect of both compounds, this delay in coagulation of about 1.3 times could have relevance in SBE. The other compounds did not interfere with the coagulant activity of *B. jararacussu* venom (Figure 1A). None of the compounds in the absence of venom clotted plasma under the experimental conditions.

Along with one arbitrary unit of the proteolytic activity of *B. jararacussu* venom (15 μg/mL), effective concentration (EC) mixed with saline solution yielded values of absorbance at approximately 0.2, which was considered as 100% of the proteolytic activity (Figure 1B). Then, one EC of *B. jararacussu* venom was incubated with each of the compounds (75 μg/mL) and DMSO (0.9% *v*/*v*, final concentration), and the inhibitory profiles of the compounds were as follows: AM50 (60%), AM51 (40%), AM52 (25%), AM54 (25%), AM55 (30%), AM56 (20%), and AM57 (40%); compound AM53 had no effect on the proteolytic activity of *B. jararacussu* venom (Figure 1B). DMSO (at the final concentration of 0.9%) under the experimental conditions did not interfere with the proteolytic activity of *B. jararacussu* venom (Figure 1B). Moreover, none of the compounds in the absence of venom hydrolyzed azocasein; thus, the compounds were devoid of proteolytic activity.

### 3.2. Effect of 1,2,3-Triazole Compound on the In Vivo Toxic Activities of B. Jararacussu Venom

**Figure 2:**
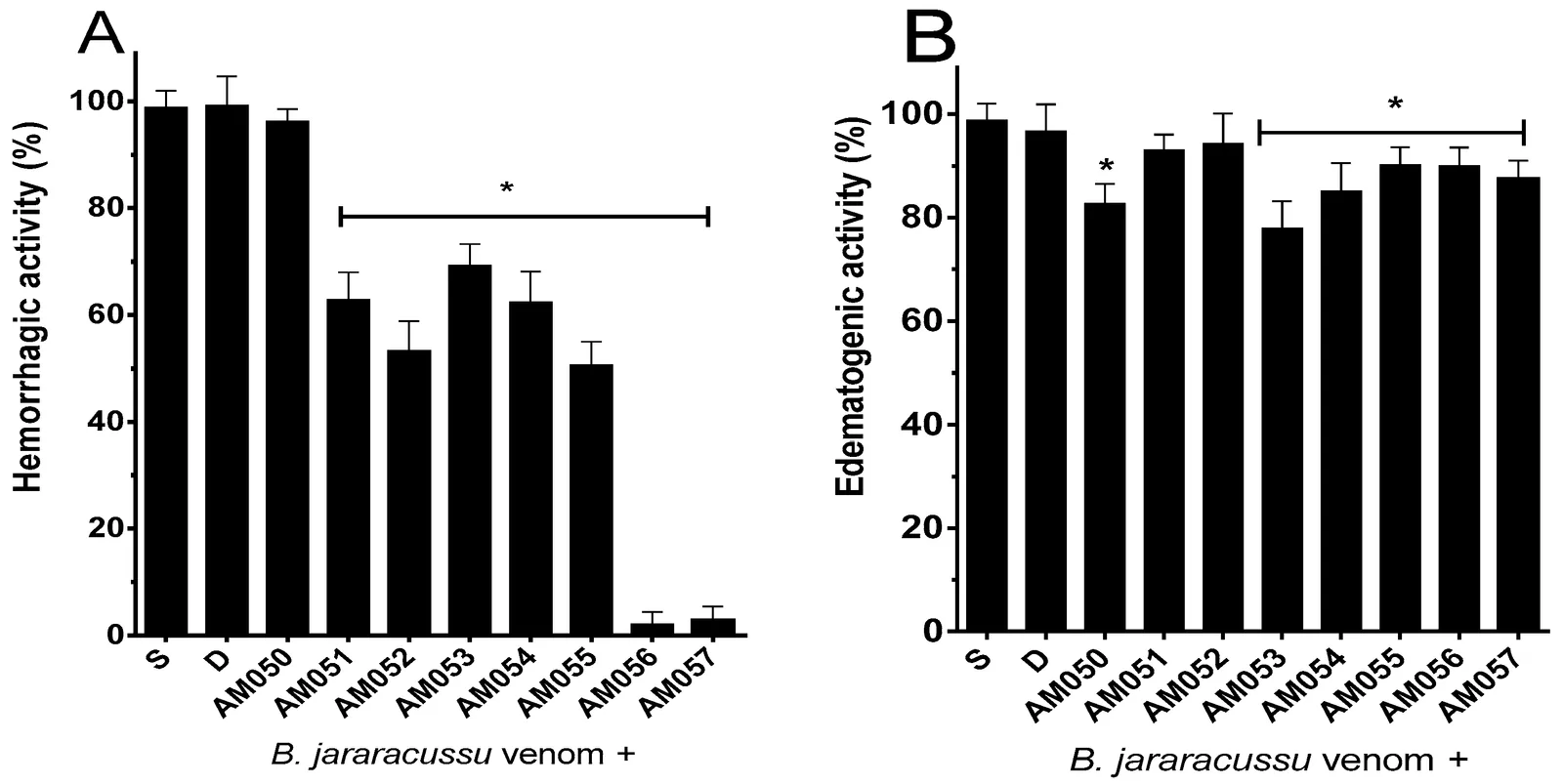
*B. jararacussu* venom was incubated with saline solution (S), DMSO (D), and AM50, AM51, AM52, AM53, AM54, AM55, AM56, and AM57 for 30 min at 25 °C. After, each mixture was injected into the mice, and the hemorrhagic (**A**) and edematogenic activities (**B**) were assessed. Results are expressed as the means ± SD (*n* = 6). * *p* < 0.05 compared with *B. jararacussu* venom + S and *B. jararacussu* venom + D.

The actions of 1,2,3-triazole compounds AM50–AM57 were assessed on the hemorrhagic (Figure 2A), edematogenic (Figure 2B), and lethal (Table 1) activities of *B. jararacussu* venom using the ratio of 1:5 venom-to-compound. However, this ratio was chosen as a screening approach and has limitations for clinical application. The injection of 2 MHD of *B. jararacussu* venom (64 μg/animal) produced a hemorrhage halo of 20 mm that was considered as 100% of hemorrhagic activity (Figure 2A). The DMSO solution did not interfere with the hemorrhagic activity of *B. jararacussu* venom (Figure 2A). As seen in Figure 2, AM50 (320 μg/mL) did not inhibit hemorrhaging by the venom, while the other compounds affected this activity with different efficacies. Compounds AM56 and AM57 fully inhibited the hemorrhagic activity of *B. jararacussu* venom, while AM51, AM52, AM53, AM54, and AM55 inhibited 30–50% the hemorrhagic activity (Figure 2A). Injection of *B. jararacussu* venom (16 μg/animal) mixed with saline solution produced an increase in paw volume that was considered as 100% of edematogenic activity (Figure 2B). Then, this dose of venom was incubated with each of the compounds (80 μg/animal), and edema was evaluated. Compounds AM51 and AM52 failed to inhibit the edema, while AM50 and AM53 inhibited 20% and compounds AM54, AM55, AM56, and AM57 approximately inhibited 10% of *B. jararacussu* venom-edematogenic activity (Figure 2B). Although inhibition percentages were 10% and 20%, this statistical difference would not imply any pharmacological relevance. However, this inhibitory action of the compounds, even if moderated, should be considered as a promising result, as preventing any toxic activity of envenomation is relevant, regardless of the percentage achieved. None of the compounds nor solvents in the absence of venom produced edema or hemorrhages in the mice.

The intraperitoneal (i.p.) injection of *B. jararacussu* venom (130 μg/animal) incubated with saline solution or DMSO (0.9% *v*/*v*, final concentration) killed the mice approximately at 65 min (Table 1). Then, this dose of venom was incubated with 160 μg of each of the compounds or a commercial antivenom for 30 min at 25 °C; the mixtures were injected into the mice and the survival times were recorded. In this assay, the 1:5 venom-to-compound ratio was not employed, as large amounts of compounds would be necessary and would lead to an increase in the concentration of DMSO, which would interfere with the proteins of the venom. Table 1 shows that the compounds AM50–AM57 prolonged the survival time of the mice, and AM57 and antivenom (AV) fully protected the mice from the lethality of *B. jararacussu* venom. The injection of compounds and antivenom in the absence of *B. jararacussu* venom did not kill the mice under the experimental conditions. The maximal period of observation of the survival time of the mice was 360 min; after, the mice were euthanized, as described in methods.

### 3.3. Predicted Toxicity and Physico-Chemical Properties of 1,2,3-Triazole Compounds

The toxicity of compounds AM50–AM57 was predicted via the OSIRIS explorer program by analyzing the parameters of absorption, distribution, metabolism, excretion, and toxicity (ADMET). As a result of this analysis, scores 1, 2, and 3 were designed for low, medium, and high potential toxicity risks, respectively. Table 2 shows that the chemical structure of all compounds had a score of 1 on tumorigenicity, which indicates no potential to cause cancer; at the reproductive effect parameter, excluding AM56 which had a score of 3, the other compounds had score values of 1 and therefore have no potential to affect fertility or cause developmental issues in the reproductive system (Table 2). Regarding the mutagenic parameter that is related to genetic damage (mutations), compounds AM50–AM54 had scores of 1, while AM55–AM57 had scores of 2. Finally, with the irritant parameter that represents potential to cause skin or eye irritation, all compounds had scores of 2 (Table 2). Therefore, the chemical structure of most of compounds had low or no alerts for predicted toxicity alerts, demonstrating favorable behavior in drug design through this in silico model.

Physicochemical and pharmacokinetic parameters of AM50–AM57 were predicted through the program SwissADME (Table 3). In the literature, in silico models are frequently employed in drug discovery, enabling the prediction of drug biological targets and drug efficacy and toxicity. As seen in Table 3, compounds AM50–AM57 have molecular weights (MW) from 173 to 219 Da, in accordance with Lipinski’s rule of five (RO5), which determine that molecules should have a molecular weight (MW) below 500 da to have good absorption and permeability. The compounds AM50–AM57 have predicted values of 5, 10, and 90 Å^2^ for HBD, HBA, and PSA, respectively (Table 3), reflecting good bioavailability that would enable compounds to easily cross lipid membranes and interact with biological targets, as well as favorable values of Caco-2 and BBB that are related to permeability and oral absorption through the gastrointestinal tract. Based on Table 3, the chemical structures of AM50–AM57 do not match with already known molecules, reinforcing the unprecedented nature of such molecules. Drug score combined with other theoretical parameters, including druglikeness, cLogP, LogS, MW, and toxicity risks, gives a single value ranging from zero to 1 that is useful to judge the overall potential of a molecule to become a drug. As seen in Table 3, most of the compounds, except AM53 and AM54, had favorable drug scores and thus met the pharmacokinetic parameters to qualify their chemical structure features for further in vitro and in vivo assays. Compounds AM50–AM53 and AM55–AM57 did not have any effect on CYP450 1A2 and CYP450 2C9, while AM54 had an effect only on CYP450 2C9 (Table 3). CYP450 1A2 and CYP450 2C9 are members of the cytochrome P450 (CYP450) super family of enzymes that is considered the most important system in the metabolism of xenobiotics, also participating in the physiological and pathological processes of the human body. Compounds AM50–AM57 had favorable predicted results regarding oral bioavailability, including BBB, LogS, and Caco2 permeability; thus, they may have good entry into cells. These parameters are related to absorption and distribution into the blood, which may influence the efficacy of a drug. In addition, these compounds had no major predicted toxicity alerts on the parameters of carcinogenic, hepatotoxic, Ames toxicity, and HERG (Table 3); the latter may affect heart function, leading to sudden death. In addition, toxicity was also evaluated through the parameters of fish toxicity and acute oral toxicity, in which results were predicted without needing to use animal models (Table 3). The compounds AM50–AM57 were labeled in category III which denotes taking caution out of safety, instead of being fatal or harmful. Finally, through in silico analysis, most of the compounds met the predicted requirements for further preclinical trials; however, it is not a guarantee of the efficacy of safety. The risk posed by compounds AM50–AM57 is difficult or impossible to assess without reliable in vitro and in vivo extrapolation. Additionally, prediction of toxicological properties through ADMET are not sufficient to establish safety of compounds or pharmacokinetic suitability, and additional approaches to better characterize toxicity should be considered, including in vivo and in vitro assays.

## 4. Discussion

Snake venom is a complex mixture of active proteins and peptides with a unique chemical composition that causes a wide range of toxic effects in victims, as previously reported in the Introduction [1,2]. Globally, SBE is a health concern of poorer regions with limited access to health care, surveillance systems, and antivenoms availability; as a consequence, millions of lives have been lost [5,21]. Recent works have described the use of small molecules with theoretical advantages over the available antivenom therapeutics, including higher stability and less adverse effects [41,42,43,44,45,46,47], which are similar features of AM50-AM57 compounds. Moreover, other drugs have been repurposed for SBE, including the PLA_2_ inhibitor varespladib [45,48] and 2,3-dimercapto-1-propane sulfonic acid (DMPS), which were able to protect envenomated animals through in vivo models [44,49]. Inhibitors of the matrix metalloproteinases (MMPs), including marimastat, prinomastat, and batimastat, are able to react with the Zn-binding motif of SVMPs, leading to inhibition of the procoagulant activity of venoms as well as lethality [49]. Batimastat and varespladib inhibited various snake venoms of medical importance and have thus undergone phase I and II clinical trials for SBE indication [50]. Previous work has identified many antivenom drugs through high-throughput screening (HTS) campaigns with potent action against SVMPs of different snake venoms; the structure–activity relationship (SAR) has also been explored [44,46,47].

Our research group assessed the triazole scaffolds specifically as inhibitors of toxic activities of snake venoms, including *B. jararaca*, *B. jararacussu*, *B. neuwiedi*, and *Lachesis muta* [30,31,32,36,37,51]. Thus, with the aim of continuing to analyze such structures in antivenom, in the present work, eight 1,2,3-triazole compounds AM50–AM57 were synthesized and characterized by ^1^H and ^13^C NMR and FTIR spectroscopy tools, and we evaluated their ability to inhibit the plasma coagulant, proteolytic, hemorrhagic, edematogenic, and lethal activities of *B. jararacussu* venom, which is a species of medical interest in the Americas. These toxic activities produced the most prominent symptoms of *Bothrops* spp. Envenomation, causing mortality and morbidity of victims, and the important family, SVSPs, SVMPs, and PLA_2_s, have been associated with such systemic and local toxic effects [11,13,14,15]. These highly toxic enzymes of snake venoms have multiple effects and broad substrate specificity, including the proteins of the coagulation cascade and platelet aggregation events, and thus may cause an imbalance in the hemostasis process [4]. SVMPs and PLA_2_ are known to be dependent on Zn^+2^ and Ca^+2^ to express their toxic activities. Moreover, the catalytically active PLA_2_s have conserved aspartic acid and histidine at positions 49 (Asp49) and 48 (His48), respectively, that interact with other amino acid residues, including glycine and tyrosine of the Ca^+2^-binding loop of the active site of PLA_2_ enzymes [15]. Therefore, AM50–AM57 may bind to Zn^+2^ and Ca^+2^ as well as critical amino acid residues in their structure, interfering with the enzymatic activity of SVMPs and PLA_2_s. SVSPs have a common mechanism of catalysis, which is the amino acid serine at position 195 (Ser195), as well as His and Asp [52,53]. Thus, the chemical groups of the compounds AM50–AM57 may also react with such amino acid residues, leading to the inhibition of the toxic activities of SVSPs, including plasma coagulant and hemorrhagic activity. However, molecular docking studies and the use of purified enzymes of *B. jararacussu* venom would help us to better understand the inhibitory mechanism of action of 1,2,3-triazoles of the present work. The work of Guan [54] described the participation of the nitrogen atom of triazoles in binding metals, and this ability leads to the formation of stable complexes with enzymes through binding to metals. In silico approaches have been employed to predict toxicity, resulting in valuable physicochemical and pharmacokinetic parameters in drug design [55,56]. Compounds derived from triazole scaffolds have been excessively investigated due to their wide range of beneficial pharmacological effects in human health, including antitumor [57], anti-leishmania and antimalarial [5], and antimycobacterial effects [35], and favorable physicochemical and economic features to become medicines, arousing interest in huge pharmaceutic industries [58,59]. Undoubtedly, the toxicologic outcomes achieved by in silico models do not guarantee success of safety and cannot be fully extrapolated in vitro and in vivo. Nevertheless, computational models are growing and may soon give a toxicological profile with no need of reagents, supplies, and animals.

Previous reports described similar 1,2,3-triazole compounds devoid of in vitro and in vivo toxicity as analyzed through the hemocompatibility and acute toxicity assays, respectively, as well as in silico tools [30,37]; these tests have been regularly employed to assess the toxicity of a molecule candidate as a medicine, providing data about systemic toxicity to cells and organs [30]. The compounds AM50–AM57 have low or no predicted parameters through in silico analyses, including mutagenicity, tumorigenic, carcinogenic, and negative effects on the reproductive system, as well as other desirable pharmacokinetic parameters of medicinal chemistry, including low MW, HBD, HBA, PSA, Caco-2 permeability, BBB, and drug score. It is difficult to postulate a single compound of this work as the most effective antivenom molecule, due to the complexity of snake venom chemical composition, as well as the widespread toxic activities caused by venoms. Therefore, inhibiting all bioactive compounds in snake venoms with a single molecule is extremely challenging. Thus, from our perspective, the use of a mixture containing the most effective 1,2,3-triazole compounds is reasonable. However, the choice of compounds is not an easy task since, as mentioned before, there are many enzymes in venoms to be neutralized; in addition, they have complex and intriguing structures and catalytic mechanisms. Nevertheless, compound AM57 could be the leading solution, as its efficacy of inhibiting coagulation in the absence and presence of compound was 65 s and 81 s, respectively, regarding proteolytic (40%), hemorrhagic (100%), and lethal (100%) activities of *B. jararacussu* venom.

On the other hand, AM57 inhibited edematogenic activity approximately by only 10%, as most of the others did. Compound AM56 could also be considered as an effective inhibitor. In addition to using a cocktail with the most active compounds, another interesting strategy could be to increase the efficacy of neutralizing the effect of venoms by combining the most active compounds with available antibothropic serum. An experiment was previously conducted by combining thiophene-triazole hybrid compounds with antibothropic serum, and this experimental procedure enhanced the inhibitory effect of the coagulant activity of *B. jararaca*, *B. jararacussu*, and *B. neuwiedi* venom more efficiently than when both were assessed separately [60]; other works [61,62,63] in which the mixture was created with the lambda carrageenan of the red alga *Chondrus crispus* [61], the plant *Schwartiza brasiliensis* [62], and the product tannic acid [63] produced the same results. In addition, the hemorrhagic, edematogenic, and lethal activities of *B. jararacussu* and *B. jararaca* venom were inhibited in the presence of antivenom; in contrast, myotoxicity caused by these venoms was poorly neutralized. Thus, the cocktail using the most active compounds mixed with antibothropic serum would be a promising strategy to treat SBE, since none of the 1,2,3-triazole compounds of this work were identified to completely inhibit all the toxic activities of this work, including proteolytic, coagulant, hemorrhagic, edematogenic, and lethal activities. It should be noticed that 1,2,3-triazole compounds were incubated with venom, which was followed by carrying out the toxic activities, including proteolytic, coagulant, hemorrhagic, edema, and lethal activities; this preincubation model does not reflect a situation of envenomation of victims. Thus, to better demonstrate antivenom efficacy, compounds should be administered after envenomation of animals.

**Figure 3:**
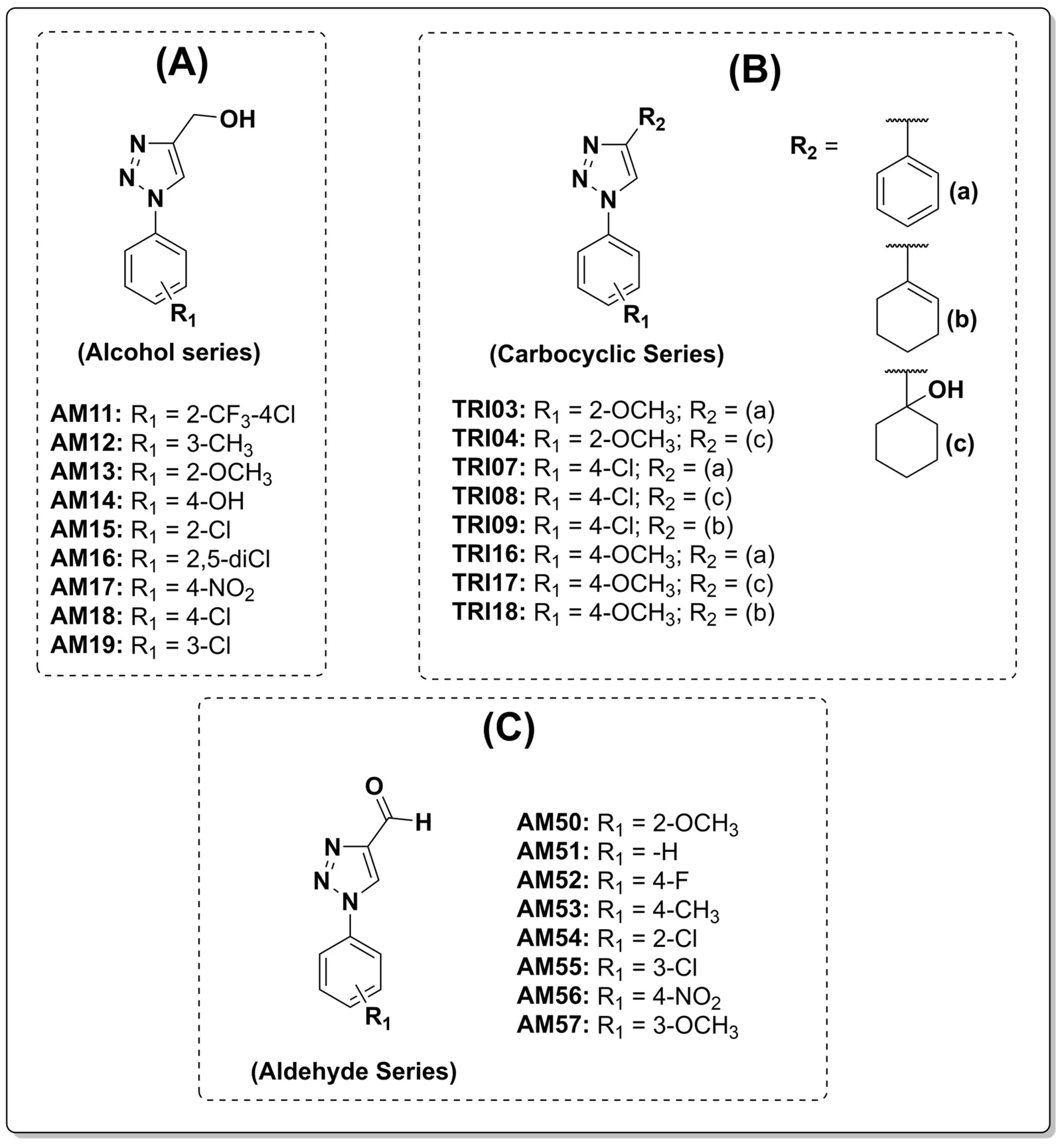
A brief comparison of the chemical structures of 1,2,3-triazoles compounds of [36] (**A**), [35] (**B**), and of this work (**C**).

Cumulative data from this work with previous results of our group [31,36,37] provide insights into a potential structural evolution of the 1,2,3-triazole scaffold (Figure 3). It is important to notice that the data of this work stem from independent studies involving different experimental protocols and snake species, including *L. muta*, *B. jararaca*, and *B. jararacussu*. However, despite variations in snake venom composition and lethality among these species, the major families of enzymes that are responsible for envenomation, including SVMPs, SVSPs, and PLA_2_s, remain evolutionarily conserved [15]. Through the analysis of the transition from carbocyclic derivatives to the current aldehydic series, we can suggest how physico-chemical parameters, including polarity and aromatic orientation, might contribute to the neutralization of the toxins of the venom of *Bothrops* spp. Moreover, the diversity of chemical groups of 1,2,3-triazole compounds, including alcohol, carboxylic, and aldehydes, have potential as inhibitors of toxins of snake venoms.

In fact, there is a correlation between the polarity of compounds and their inhibitory profile on the toxic activities of *B. jararacussu* venom. In the work of Amorim [36], compounds of the series TRI03–TRI18 (Figure 3B) and the use of bulky and apolar carbocyclic groups at R2 position favored the neutralization of proteolytic activity, which is associated with local tissue damage, hemorrhage and coagulation disturbances caused by snake venom; this suggests that high lipophilicity could facilitate interactions with hydrophobic pockets of enzymes or cell membranes.

However, achieving effective protection against hemorrhage and lethality appears to require a shift toward higher polarity. The transition to the alcohol series and, ultimately, the current aldehyde series points toward a change in the inhibition threshold. The increased electrophilic character of the aldehyde group, combined with its higher polarity, might promote a more stable interaction with the zinc-dependent catalytic site of snake venom, including SVMPs [64]. This chemical evolution may explain the leap in efficacy of some compounds of this work as inhibitors, while the previous series of [36,37] only prolonged mortality caused by the injection of *B. jararacussu* venom, but AM57 completely protected the mice from death.

The aromatic ring likely serves as a structural anchor, potentially forming π-π stacking interactions with aromatic residues in the active site of snake venom enzymes. It is interesting to observe that the methoxy group consistently emerged as a pharmacological hotspot in the lead compounds of all three series, including TRI16, TRI18, AM13, and AM57. Interestingly, the efficiency of the methoxy substitution seems to be highly dependent on its position, which might need to be precisely adjusted to compensate the changes at the R2 position and variations in the venom proteome. In the series of this work, the comparison of AM57 (3-OCH_3_) with AM50 (2-OCH_3_) and AM51 (unsubstituted phenyl ring) revealed the importance of the presence and the position of substituents. While AM50 showed promising results as an inhibitor of the proteolytic activity of *B. jararacussu* venom (60%), AM51 was ineffective; thus, the methoxy substituent should be considered important in the development of antivenom molecules. Moreover, only the specific orientation of the methoxy group within the chemical structure of AM57 provided enough fit to prevent death of the mice. Furthermore, the presence of electron-withdrawing groups, such as the nitro group in AM56, proved highly effective in fully neutralizing the hemorrhagic activity of *B. jararacussu* venom, even though lethality was not completely inhibited. The shift from *para* to *ortho* and now meta in AM57 suggests that the steric orientation of the methoxy group could be vital for achieving the fit required to interfere with the catalytic machinery of *Bothrops* toxins.

All series, including carboxylic acid, alcohol, and aldehydes particularly, effectively inhibited the hemorrhagic and proteolytic activity of *B. jararacussu* venom but exhibited a weak impact on the venom-induced edema. Compounds AM51 and AM52 failed to inhibit edema, while the others, including AM57, inhibited approximately 10–20% of that activity. This selectivity suggests that the 1,2,3-triazole core, especially when functionalized with an aldehyde, inhibited venom proteases of venom, including SVSPs and SVMPs, more effectively than PLA_2_ enzymes. In the case of *B. jararacussu* venom, this targeted inhibition of proteases appears sufficient to prevent lethality, hemorrhage, and hemostatic disturbances, even without fully neutralizing edematogenic activity. However, in this work, this observation requires experimental confirmation.

The consistent performance of some structural motifs against different toxic activities of *B. jararacussu* venom, most notably the methoxy group of AM57, TRI16, TRI18, and AM13, suggests a consistent pharmacophoric contribution rather than unrelated findings. While AM57 stands out as the lead prototype for further preclinical trials, the inhibitory behavior of the series of AM50–AM57 compounds validates the triazole scaffold core as a versatile platform for developing inhibitors of SVMPs, SVSPs, and PLA_2_s. While the structure–activity relationship (SAR) trends provide a preliminary framework and valuable tools in the drug design process, the exact binding and mechanism of action of such compounds were not addressed, and this would be a limitation of this work. In particular, other approaches could be taken to overcome these limitations, including the use of the specific proteases chromogenic substrates S-2238 and S-2288 and the lipid fluorescent NBD-PC, 1-palmitoyl-2-{6-[(7-nitro-2-1,3-benzoxadiazol-4-yl)amino]hexanoyl}-sn-glycero-3-phosphocholine, commonly employed as substrate to assess the activity of PLA_2_ enzymes. Lastly, as previously mentioned, molecular docking and testing of 1,2,3-triazole compounds against purified enzymes of *Bothrops* spp. venoms would give valuable data to solve and overcome the limitations of this work regarding the inhibitory mechanism of the compounds. Moreover, in vitro and in vivo toxicity of the compounds must be assessed, even though previous data reported that triazoles were non-toxic via such assays. Taken together, the compounds described in this manuscript may give valuable leads for further studies to design a rationale of even more potent and selective inhibitors to treat envenomation by snake venoms.

## 5. Conclusions

This study demonstrated the ability of eight triazole compounds to inhibit a set of toxic activities caused by the venom of the snake *B. jararacussu*. AM54, AM56, and AM57 may be considered the lead compounds and potential adjunctive candidates to the available antivenom as a therapeutic. However, to understand the inhibitory mechanism of the compounds, performing post-envenomation models and testing 1,2,3-triazole compounds combined with antivenoms should be considered and require further investigation to postulate such compounds as candidates for antivenoms.

## Figures and Tables

**Table 1:** Effect of 1,2,3-triazole compounds on the lethal activity of *B. jararacussu* venom.

Groups	Survival Time (min)
*B. jararacussu* venom + S	67 ± 12
*B. jararacussu* venom + D	64 ± 10
*B. jararacussu* venom + AV	360 ± 0 *
*B. jararacussu* venom + AM50	165 ± 42 *
*B. jararacussu* venom + AM51	139 ± 19 *
*B. jararacussu* venom + AM52	147 ± 41 *
*B. jararacussu* venom + AM53	94 ± 21 *
*B. jararacussu* venom + AM54	326 ± 12 *
*B. jararacussu* venom + AM55	170 ± 20 *
*B. jararacussu* venom + AM56	152 ± 33 *
*B. jararacussu* venom + AM57	360 ± 0 *

*B. jararacussu* venom was incubated with saline solution (S), DMSO (D), antivenom (AV), or compounds AM50, AM51, AM52, AM53, AM54, AM55, AM56, and AM57 for 30 min at 25 °C. Then, each mixture was injected i.p. into the mice and the survival time of the mice was registered. Results are expressed as the means ± SD (*n* = 6). * *p* < 0.05 compared with *B. jararacussu* venom + S and *B. jararacussu* venom + D.

**Table 2:** In silico toxicity of the 1,2,3-triazole compounds AM50, AM51, AM52, AM53, AM54, AM55, AM56, and AM57 according to the OSIRIS Property Explorer program.

Compounds	Mutagenic	Tumorigenic	Reproductive Effects	Skin Irritant
AM50	1	1	1	2
AM51	1	1	1	2
AM52	1	1	1	2
AM53	1	1	1	2
AM54	1	1	1	2
AM55	2	1	1	2
AM56	2	1	3	2
AM57	2	1	1	2

1 indicates low or no significant alerts for toxicity; 2 indicates medium or moderate risk; and 3 indicates a high and undesirable potential for toxicity.

**Table 3:** Toxicology and pharmacokinetics parameters of 1,2,3-triazole compounds AM50, AM51, AM52, AM53, AM54, AM55, AM56, and AM57 using prediction computational tools.

Parameters	AM50	AM51	AM52	AM53	AM54	AM55	AM56	AM57
MW	203.20	173.17	191.16	187.20	207.62	207.62	218.17	203.20
clogP	−0.91	0.07	−0.47	0.25	−0.07	−0.0	−0.62	−0.91
LogS	−1.69	−1.71	−2.42	−1.71	−3.16	−3.68	−2.42	−2.00
HBA	5	4	5	4	4	4	7	5
HBD	0	0	0	0	0	0	0	0
PSA	43.03	39.37	39.34	39.37	39.60	39.36	78.21	46.20
Druglikeness	−3.37	−2.37	−2.130	−3.11	−6.79	−2.61	−4.70	−4.32
Drugscore	0.40	0.42	0.42	0.19	0.29	0.40	0.39	0.39
Caco-2	Yes	Yes	Yes	Yes	Yes	Yes	Yes	Yes
BBB	Yes	Yes	Yes	Yes	Yes	Yes	Yes	Yes
CYP 2C9	No	No	No	No	Yes	No	No	No
CYP 1A2	No	No	No	No	No	No	No	No
BS	Yes	Yes	Yes	Yes	Yes	Yes	Yes	Yes
Carcinogenic	No	No	No	No	No	No	No	No
Ames toxicity	No	No	Yes	No	No	No	No	No
Hepatoxicity	No	No	No	No	No	No	No	No
HERG	No	No	No	No	No	No	No	No
Fish toxicity	No	No	No	No	No	No	No	No
Oral toxicity	III	III	III	III	III	III	III	III

MW, molecular weight; cLogP, lipophilicity; LogS, solubility prediction; HBA, hydrogen bond acceptors; HBD, hydrogen bond donors; PSA, polar surface area; Caco-2, related to permeability and oral absorption through the gastrointestinal tract; BBB, blood–brain barrier penetration; CYP 1A2 substrate and CYP 2C9 inhibitor, predict inhibition of cytochrome P450 isoforms; BS, Bioavailability Score, which indicates how well a substance, particularly a drug, is absorbed into the bloodstream after oral administration; HERG, human Ether-a-go-go-related, that is, related to repolarizing the action potential of cardiac muscle.

## Data Availability

The original contributions presented in this study are included in the article. Further inquiries can be directed to the corresponding author.

## Abbreviations

The following abbreviations are used in this manuscript:

WHO	World Health Organization
MCC	Minimum coagulation concentration
EU	Enzymatic unit
MHD	Minimum hemorrhage dose
SVSPs	Snake venom serine proteinases
SVMPs	Snake venom metaloproteinases
PLA_2_s	Phospholipases A_2_
DMSO	Dimethyl sulfoxide
FTIR	Fourier transform infrared
NMR	Nuclear magnetic resonance
